# The miRNA Profile of Platelets Stored in a Blood Bank and Its Relation to Cellular Damage from Storage

**DOI:** 10.1371/journal.pone.0129399

**Published:** 2015-06-29

**Authors:** Thaís Brilhante Pontes, Caroline de Fátima Aquino Moreira-Nunes, Jersey Heitor da Silva Maués, Letícia Martins Lamarão, José Alexandre Rodrigues de Lemos, Raquel Carvalho Montenegro, Rommel Mário Rodriguez Burbano

**Affiliations:** 1 Laboratory of Human Cytogenetics, Institute of Biological Sciences, Federal University of Pará, Belém, PA, 66075110, Brazil; 2 Laboratory of Genetics and Molecular Biology, Foundation Center for Hemotherapy and Hematology of Pará (HEMOPA), Belém, PA, 66033–000, Brazil; 3 Institute of Biological Sciences, Federal University of Pará, Belém, PA, 66075110, Brazil; Royal College of Surgeons, IRELAND

## Abstract

Millions of blood products are transfused each year, and many lives are directly affected by transfusion. Platelet concentrate (PC) is one of the main products derived from blood. Even under good storage conditions, PC is likely to suffer cell damage. The shape of platelets changes after 5 to 7 days of storage at 22°C. Taking into consideration that some platelet proteins undergo changes in their shape and functionality during PC storage. Sixteen PC bags were collected and each PC bag tube was cut into six equal pieces to perform experiments with platelets from six different days of storage. Thus, on the first day of storage, 1/6 of the tube was used for miRNA extraction, and the remaining 5/6 was stored under the same conditions until extraction of miRNAs on each the following five days. Samples were sequenced on an Illumina Platform to demonstrate the most highly expressed miRNAs. Three miRNAs, mir127, mir191 and mir320a were validated by real-time quantitative PCR (RQ-PCR) in 100 PC bags tubes. Our method suggests, the use of the miRNAs mir127 and mir320a as biomarkers to assess the "validity period" of PC bags stored in blood banks for long periods. Thus, bags can be tested on the 5th day of storage for the relative expression levels of mir127 and mir320a. Thus, we highlight candidate miRNAs as biomarkers of storage damage that can be used as tools to evaluate the quality of stored PC. The use of miRNAs as biomarkers of damage is unprecedented and will contribute to improved quality of blood products for transfusions.

## Introduction

In 2012, there were over 3.6 million blood donations and 3.1 million blood transfusions performed in Brazil [1]. Many lives are affected by transfusions, and there is a growing need to improve the tools and strategies that assess the quality of transfusion products during production and handling, thereby ensuring safety and efficacy for patient health [2].

Platelet concentrate (PC) is one the main components used for transfusion medicine. Platelets are small, anucleate cells derived from cytoplasmic fragmentation of megakaryocytes in the bone marrow. When stimulated, platelets participate in clotting both through their physical shape and by releasing clotting factors. Thus, they are essential for maintaining homeostasis and vascular integrity and are activated by exposed collagen in the endothelium of damaged blood vessels [3–4].

PC transfusion is an important medical procedure for patients undergoing major surgery or high-dose chemotherapy and patients with thrombocytopenia [5]. Clinical indications for platelet transfusion are to prevent or control bleeding in patients with thrombocytopenia or, less often, in patients with thrombocytopathy [6].

PC is composed of a suspension of platelets in plasma, which is prepared by double centrifugation of a unit of whole blood (WB) or by apheresis, method that selectively removes only this component from the donor [5,7]. Each component obtained from WB has ideal storage conditions that allow its specific activities and functions to be preserved [8].

Even under ideal storage conditions, modifications and/or degradation of components in blood bags may occur. Such changes, known as "storage damage", affect the useful lifespan and quality of products derived from stored blood and consist of morphological changes, platelet activation, changes to membrane glycoproteins and proteolysis and expression of platelet surface receptors [9, 10, 11, 12, 13].

MicroRNAs (miRNAs) are small, noncoding RNA molecules that are approximately 22 nucleotides long in their mature state and that are involved in regulating gene expression in the cell. This regulation occurs through the binding of the miRNA molecule to a messenger RNA with imperfect complementarity in the 3' untranslated region (UTR), thereby disrupting translation and preventing gene expression [14]. Because miRNAs can alter the level of translation without destroying the transcript, it is possible that significant changes can occur within the cell that are not detected at the transcriptome level [15,16, 17].

Edelstein et al., reported high levels of miRNAs in normal human platelets and evidence suggesting the biological and clinical relevance of these molecules, where beyond controlling gene expression, miRNAs would play an important role as biomarkers of hematological diseases and platelet reactivity and serve as a tool for understanding the mechanisms of gene expression in platelets [18].

To date, there are no reports in the literature characterizing the set of miRNAs expressed (miRnome) in PC after storage in blood banks. Therefore, the present study characterized the most highly expressed miRNAs in PC using high coverage sequencing, examined quantitative changes in miRNA levels during and after storage in a blood bank, then validated by real-time quantitative polymerase chain reaction (RQ-PCR) two miRNAs in 100 PC bags tubes and aimed to propose candidate miRNAs as biomarkers of PC storage damage. This study is novel.

## Materials and Methods

### Collection and Processing of Platelet Samples

Buffy coat, the traditional approach for obtaining PC, was used in this experiment [19, 20]. To obtain PC, a bag of WB (Bag-Matrix, 450 ml) was centrifuged (Thermo Scientific) and fractionated on a Compomat G5 hematologic processor (Fresenius Kabi), which, after separating platelets from red blood cells, automatically deposited the PC in a satellite bag [21] where 99,9% (>3 log10) leukocytes were removed by filtration [22]. Next, a PC bag was established by pooling the PC from five satellite bags of healthy volunteers with the same blood type and Rh factor with negative serological results for blood-borne diseases provided by the Foundation Center for Hemotherapy and Hematology of Pará (Fundação Centro de Hemoterapia e Hematologia do Pará —HEMOPA).

For this study, PC bags containing 6.0 x 10^10^ platelets per bag were selected. In this work, the PC were stored in the same conditions as used in PC transfusion, that is optimal conditions. The quality control of PC bags was assessed at HEMOPA Foundation, using the following parameters: swirling, volume of the platelet concentrate, WBC count, platelet count and pH. For this experiment, each of the six days tested, two bags PC tubes were subjected to routine quality control tests and the results were similar to the PC bags used in blood transfusions (Table 1).

**Table 1:** Quality control of platelet concentrate bag tubes.

Parameters	1 day	2 day	3 day	4 day	5 day	7 day
Swirling	Positive	Positive	Positive	Positive	Positive	Positive
Volume of the platelet concentrate (18 ml by PC bags tube).	3 ml	3 ml	3 ml	3 ml	3 ml	3 ml
WBC count (10^8^/ 70ml)	0,51 ± 0,02	0,48 ± 0,02	0,42 ± 0,03	0,34 ± 0,03	0,25 ± 0,04	0,12 ± 0,06
Platelet count (10^10^)	7,20 ± 0,10	6,9± 0,11	6,7 ± 0,15	6,4 ± 0,18	6,1 ± 0,21	5,5 ± 0,30
pH	7,2 ± 0,00	7,2 ± 0,01	7,2 ± 0,01	7,3 ± 0,02	7,3 ± 0,03	7,4 ± 0,03

The method of preparing PC bags used in this study was performed following the criteria of Ordinance No. 1,353 from June 13, 2011 of the Brazilian Ministry of Health and was conducted within the first 8 hours after collection of WB in a closed circuit; the first RNA extraction was performed after 24 hours of storage [23]. Thus, "newly collected platelets" correspond to the first day of storage at the HEMOPA Foundation. Sixteen PC bags were collected; four were blood type A, 11 were type O, and one was type B. All bags were positive for Rh factor. As each PC bag consisted of platelets from five donors, this project analyzed platelet miRNAs from 80 donors in total.

### PC Collection and microRNA Extraction to Solexa Sequencing

The upper edge of the PC bags had a tube or cord measuring approximately 38 cm that was filled with platelets and was removed from the PC bags to be used in this experiment. This tube or cord is comprised of the same plastic material "breathable" of the bags, which allows for maximum oxygen diffusion. Thus, the bag of remaining platelets was utilized as usual in blood transfusions without burdening the HEMOPA Foundation. Since the amount of extracted miRNA from one PC bag tube is not sufficient to perform a new individual generation sequencing, this experiment was performed using pools PC.

Each PC bag tube was cut into six equal pieces to perform experiments with platelets from six different days of storage (1st, 2nd, 3rd, 4th, 5th and 7th). Thus, on the first day of storage, 1/6 of the tube was used for miRNA extraction, and the remaining 5/6 was stored under the same conditions as PC bags at 22 ± 2°C under constant agitation until extraction of miRNAs on each the following five days.

On each of the six days of storage, miRNA was extracted from 16 fragments of different PC bag tubes. As the PC is stored in the blood bank for a period ranging from three to five days, depending on the plasticizer in the storage bag [19], the 1st day is the high-quality control for platelets, and the 7th day is the low-quality control for platelets.

The mirVANA miRNA isolation kit (Life Technologies) was used to extract miRNA following the protocol suggested by the manufacturer. After analyzing and quantifying RNA integrity, homogeneous aliquots from the 16 extractions from each day of the experiment were mixed in a single tube. Thus, when extractions were completed, six pools were isolated consisting of PC miRNAs from 80 donors participating in the experiment but for six different lengths of storage (PC from the 1st, 2nd, 3rd, 4th, 5th and 7th days of storage).

### PC Collection, miRNA Extraction and miRNA expression validation

To validate the results obtained by Solexa sequencing, which were performed on pools of PC, 100 different PC bags tubes were used, randomly chosen, which also was cut into six equal pieces to perform experiments with platelets from six different days of storage (1st, 2nd, 3rd, 4th, 5th and 7th). Thus, on the first day of storage, 1/6 of the 100 tubes was used for miRNA extraction, and the remaining 5/6 of 100 PC bag tubes was stored under the same conditions as PC bags at 22 ± 2°C under constant agitation until extraction of miRNAs on each the following five days. On each of the six days of storage, miRNA was extracted from 100 fragments of different PC bag tubes, totaling 600 fragments.

The mirVANA miRNA isolation kit (Life Technologies) also was used to extract miRNA of 100 different PC bags tubes. As each PC bags tubes consists of five platelet donors, 500 donors were used to this validation experiment.

### Quantification of RNA

Quantitation was performed using the Qubit Fluorometer (Invitrogen) spectrophotometer following the protocol recommended by the manufacturer. The quality of total RNA was measured by the amplification quality of the endogenous β-actin gene (ACTB).

### Preparation of cDNA libraries

The cDNA libraries were constructed using the TruSeq Small RNA Sample Prep Kit (Illumina) following the protocol recommended by the manufacturer. The process is summarized by the binding of adapters to the 3' and 5' regions of the RNA molecule. The 3' adapter is specially modified to bind to the 3'-hydroxyl group of miRNAs and other small RNAs resulting from processing by Dicer. After adapter binding, a reverse transcriptase reaction synthesizes single-stranded complementary DNA (cDNA). The cDNA is then amplified by PCR using a common primer and a primer containing an index sequence in two separate and subsequent readings.

### Analysis of RNA Integrity

RNA quality was determined by the RNA integrity number (RIN) using the Bioanalyzer 2100 (Agilent Technologies) platform. The RIN is an estimate of the integrity of total RNA samples.

#### Sequencing on the Illumina Platform

Libraries were sequenced on an Illumina GA IIx (SCS 2.8 software; Illumina, San Diego, CA, USA), with a 32-mer single-end sequence. Image analysis and base calling were performed using the RTA 1.8 software.

### Processing of Sequencing Runs

The files generated by sequencing, with the.fastq extension, were converted to double encoder format and imported into the CutAdapt software (version 0.9.4) for the removal of adapters and conversion to the.fastq format, which is the input format for the alignment program. The FASTQC software (version 0.9.2) was used to assess read quality.

The latest version of the human genome (Hg19/GRCh37), as provided by The Genome Reference Consortium present in the database of the National Center for Biotechnology Information (NCBI), was used as a reference for the alignment of sequenced libraries. The Spliced Transcripts Alignment to a Reference (STAR) program was used to align sequences [24]. MiRNAs generated by sequencing were identified according to the most recent miRNA database, miRBase version 20 (http://www.mirbase.org/).

All miRNAs sequences of platelets are available in the NCBI Gene Expression Omnibus (GSE61856).

### Analysis of Expression Data

To normalize the gene expression data values generated by mapping, the reads, in "reads per million" (RPM), were calculated in logarithm base 2 or binary logarithm (log2). The DEGseq program (version 1.17.1) present in the R analysis package was used to analyze the differential expression profiles of platelet miRNAs at each of the six different storage timepoints (1st, 2nd, 3rd, 4th, 5th and 7th days of storage). This program identifies differentially expressed genes or isoforms from data generated by the sequencing of RNA in different samples [25]. The R analysis package was also used to generate graphs.

### MiRNA expression validation

To quantitate miRNA levels of mir-127 and mir-320a, the miRNA was isolated from 100 PC bags tubes using mirVanaTM miRNA isolation kit (Ambion). The miRNA was reverse transcribed using the TaqMan MicroRNA Reverse Transcription kit according to the manufacturer’s protocol (Life Technologies, USA) with TaqMan microRNA Assays for mir-127 (PN002229), mir-320a (PN002277) and mir-191 (PN002678) to RQ-PCR. Complementary DNA was then amplified by real-time PCR using the TaqMan Universal Master Mix II with UNG (Life Technologies, USA) on a Rotor-Gene Q (Qiagen, Germany). mir-191 was selected as an internal control for miRNA input and reverse transcription efficiency because was the miRNA of most highly expressed on six different days of storage. All RQ-PCR reactions were performed in triplicate for both miRNA.

## Results

### Expression Profile of miRNAs in Freshly Collected Platelets

Sequence analysis and identification of miRNAs were performed according to the miRBase database (version 20), which to date contains information for 1870 registered miRNAs. Table 2 summarizes the number of miRNAs identified on the six days of PC storage in the HEMOPA Foundation. On the 1st day of PC storage, which represents the high-quality control for platelets, 675 miRNAs were identified. On the 7th day of PC storage, which corresponds to the low-quality control for these cells, 579 miRNAs were identified. However, the fewest miRNAs, 526, were detected on the fifth day of storage.

**Table 2:** Number of miRNAs identified in the platelet concentrate (PC) according to miRBase version 20 on different days of storage.

Days of PCs Storage							
*Periods	1^st^	2^nd^	3^rd^	4^th^	5^th^	7^th^	Total
microRNAs	311	308	315	312	332	321	1899
(% microRNAs)	46.21	45.77	46.81	46.36	49.33	47.70	—-
(Reads ≥ 10)	1.272.181	1.317.056	1.234.576	1.441.070	1.121.352	1.274.906	6.344.086

*days

To define the differential expression profile of the platelet miRnome, sets of miRNAs expressed in the PC bags from the 2nd to 7th day of storage were compared with those from the 1st day. The identified miRNAs were considered differentially expressed when the expression difference reached two-fold (2X) with p ≤ 0.05 (Table 3).

**Table 3:** Number of microRNAs highly expressed in platelet concentrate (PC), according to days of storage.

MicroRNA mature sequences detected in human platelets								
miRBase Database		Deep Sequencing (Raw_Reads)						
Platelets miRNAs	Mature Sequence	(nt)	1^st^	2^nd^	3^rd^	4^th^	5^th^	7^th^
let-7b	5p-UGAGGUAGUAGGUUGUGUGGUU	22	76258.72	93880.88	81166.40	232478.10	154268.94	209761.63
let-7g	5p-UGAGGUAGUAGUUUGUACAGUU	22	30660.67	50650.96	31569.77	47051.60	32495.43	41100.61
let-7i	5p-UGAGGUAGUAGUUUGUGCUGUU	22	153624.39	177540.69	187708.74	156104.61	112702.28	158197.45
miR-127	5p-CUGAAGCUCAGAGGGCUCUGAU	22	53873.53	82979.84	79817.73	40357.34	27041.92	33505.19
miR-191	5p-CAACGGAAUCCCAAAAGCAGCUG	23	389782.51	327464.40	306686.45	277301.57	207491.82	212457.63
miR-22	5p-AGUUCUUCAGUGGCAAGCUUUA	22	30309.21	32351.18	26765.51	20082.79	44801.51	36856.04
miR-221	5p-ACCUGGCAUACAAUGUAGAUUU	22	28508.84	34872.78	34905.24	22588.03	21286.25	22400.77
miR-320ª	AAAAGCUGGGUUGAGAGGGCGA	22	52054.80	60729.93	76080.13	89440.35	67412.33	81753.13
miR-423	5p-UGAGGGGCAGAGAGCGAGACUUU	23	34337.39	46224.94	51367.02	71444.64	59088.24	60370.46
miR-99b	5p-CACCCGUAGAACCGACCUUGCG	22	28068.09	7844.89	9183.39	5535.05	5610.80	5743.72

### Degradation profile of miRNAs

**Fig 1:**
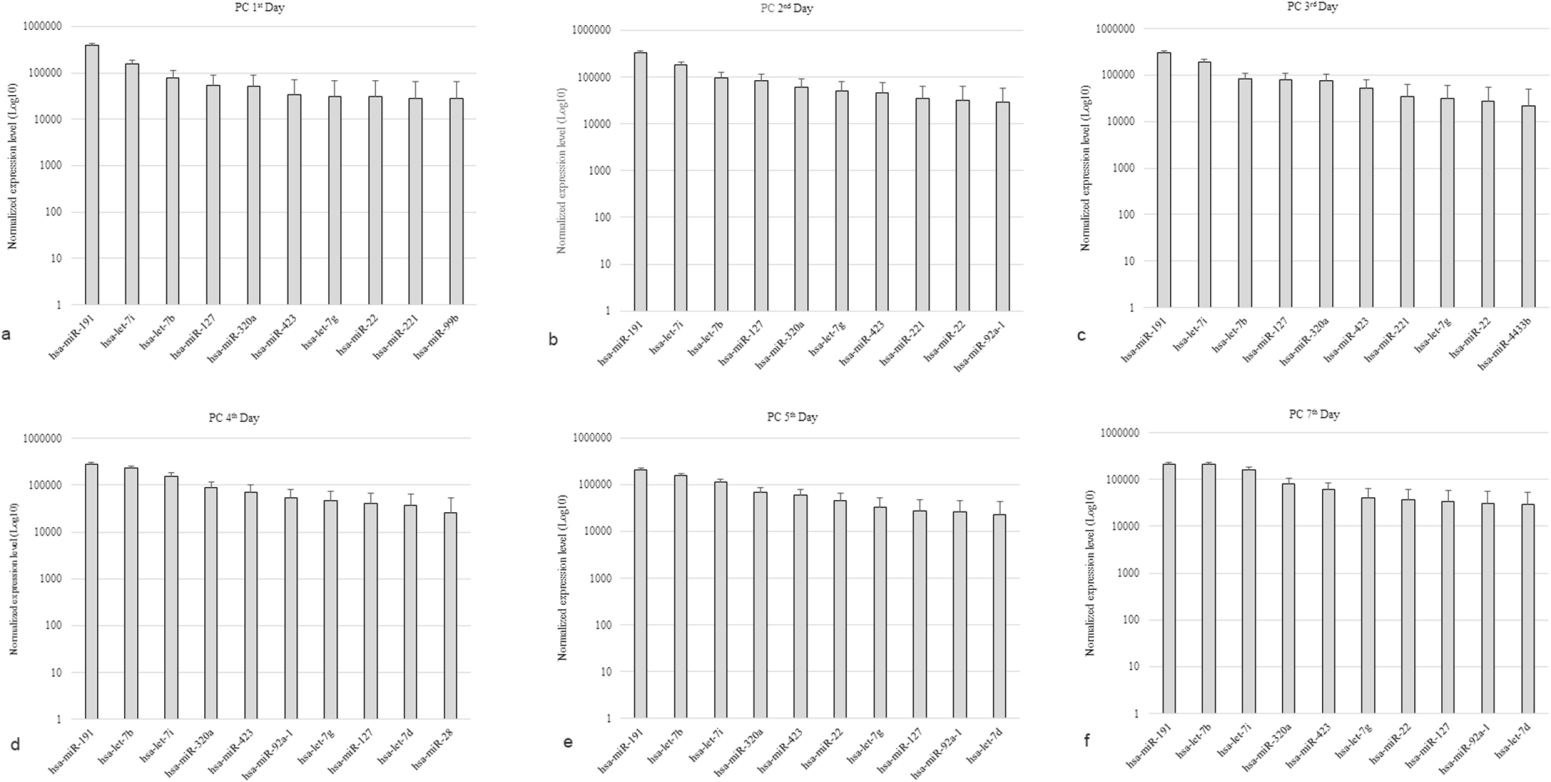
Expression profile of most highly expressed miRNAs, in log10 scale, in PCs on each day of storage. (A) Profile of the ten most expressed miRNAs on the 1st day of storage, considered as positive control; (B) Profile of the ten most expressed miRNAs on the 2nd day of storage with nine of the ten miRNAs present on the first day, differing only by miR-92a-1 in place of miR-99b; (C) Profile of the ten most expressed miRNAs on the 3rd day of storage, with nine of the ten most expressed miRNAs in the 1st and 3rd day; (D) Profile of the ten most expressed miRNAs on the 4th day of storage, with seven of the ten most expressed miRNAs on Day 1, differing by miRNAs let-7d, miR-28 and miR-92a-1;(E) Profile of the ten most expressed miRNAs on the 5th day of storage, with eight of the ten most expressed miRNAs on Day 1, with the exception of miR-92a-1 and let-7d; (F) Profile of the ten most miRNAs expressed in the 7th day of storage are similar to day 5, with quantitative variation. Errors bars show standard error of expression levels by reads per million.

The quantitative loss of the 10 miRNAs with highest expression in the PC from the 1st to the 7th day of storage was analyzed. Expression was ranked based on raw RPM expression values. The 10 miRNAs with the highest expression on day 1 of PC storage, in descending order, were hsa-mir-191, hsa-let-7i, hsa-let-7b, hsa-mir-127, hsa-mir-320a, hsa-mir-423, hsa-let-7g, hsa-mir-22, hsa-mir-221 and hsa-mir-99b (Fig 1A).

On the 2nd day of PC storage, the expression profile featured nine of the 10 miRNAs most highly expressed on day 1 because miRNA hsa-mir-99b, which corresponds to the 10th most highly expressed miRNA, quantitatively decreased and miRNA hsa-mir-92a-1 replaced it among the 10 most highly expressed miRNAs (Fig 1B).

On the 3rd day of PC storage, the expression profile featured nine of the 10 most highly expressed miRNAs from the 1st and 2nd days because the 10th most highly expressed miRNA on the 2nd day of storage, hsa-mir-92a-1, quantitatively decreased and another miRNA, hsa-mir-4433b, replaced it among the 10 most highly expressed miRNAs (Fig 1C).

Analyzing the expression profile of miRNAs in PC on the 4th day of storage revealed that the miRNAs hsa-mir-221, hsa-mir-22 and hsa-mir-4433b, which were among the 10 most highly expressed miRNAs in PC between the 1st and 3rd days of storage, quantitatively decreased and were replaced on the 4th day of storage by miRNAs hsa-mir-92a-1, hsa-let-7d and hsa-mir-28 (Fig 1D).

A comparison of the PC from the 1st and the 5th days of storage revealed eight miRNAs in common because the 9th and 10th most highly expressed miRNAs from the 1st day of PC storage, hsa-mir-221 and hsa-mir-99b, respectively, began to quantitatively decline on day 5 and were replaced by miRNAs hsa-mir-92a-1 and hsa-let-7d. Additionally, starting on the 4th day of storage, the miRNA hsa-mir-28 quantitatively decreased and was replaced by the miRNA hsa-mir-22 on the list of the 10 most highly expressed miRNAs on the 5th day of storage (Fig 1E).

The expression profiles of the 10 most highly expressed miRNAs in PC were similar on the 5th and 7th days of storage, with quantitative variations, but the miRNAs occurred in the same order from the most highly expressed miRNA (hsa-mir-191) to the miRNA with lowest expression (hsa-let-7d) (Fig 1F).

**Fig 2:**
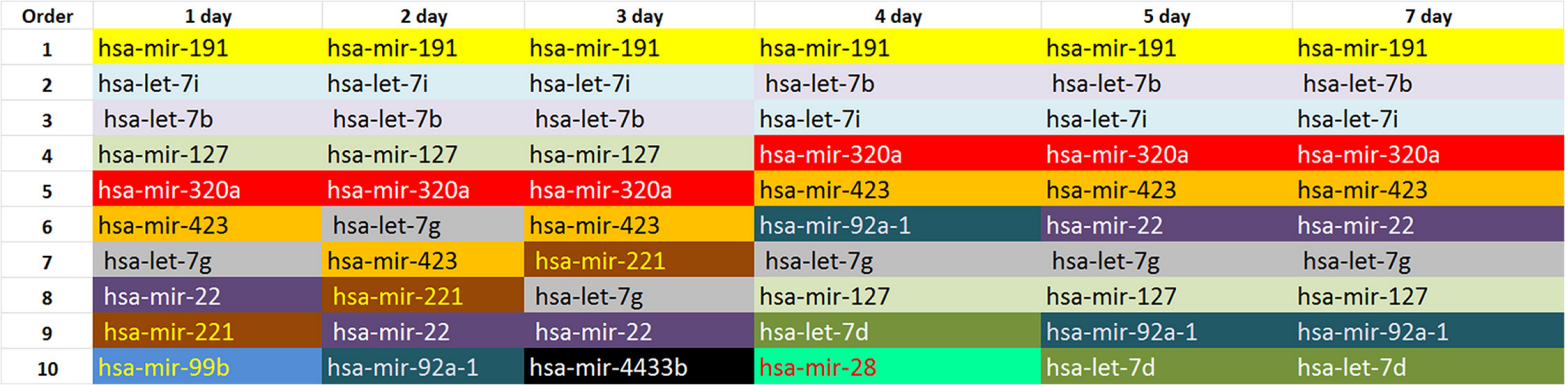
List of the 10 most highly expressed miRNAs, in descending order, on the six days of platelet concentrate storage. In total, 14 miRNAs were included in the six lists.

**Fig 3:**
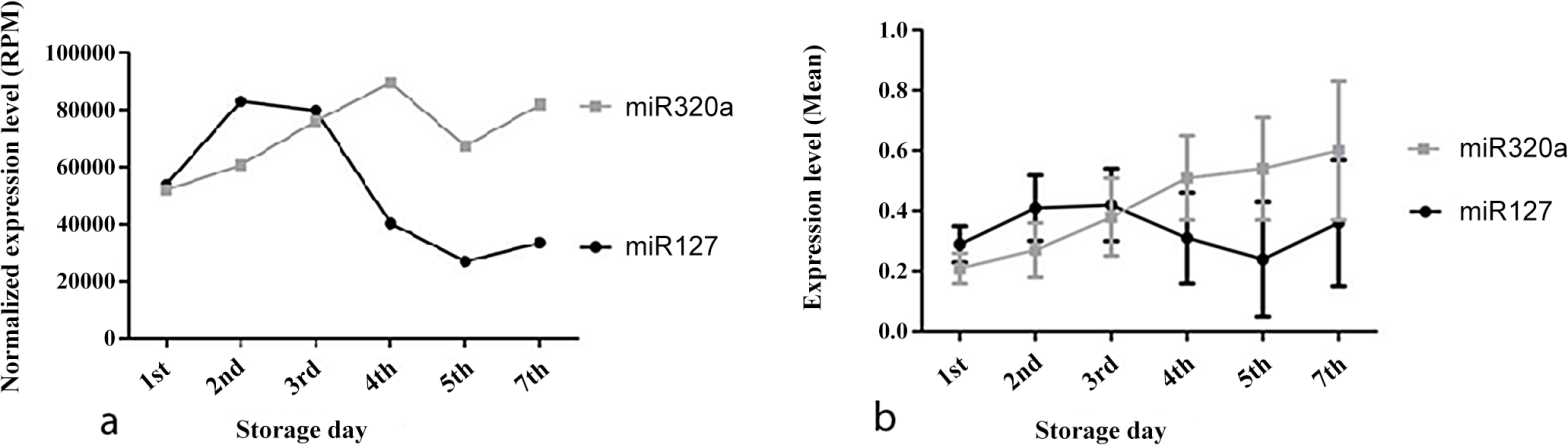
Expression profile of miRNAs hsa-miR-127 and hsa-miR-320a in PC bags during the six days of storage investigate. (A) Expression profile in reads per million of miRNAs miR-127 and miR-320a; (B) Average expression of miRNAs miR127 and miR320a and their respective standard deviations.

Fig 2 summarizes the list of the 10 most highly expressed miRNAs on the six days of PC storage. In total, 14 miRNAs are present on the six lists. The miRNA hsa-mir-191 was the most highly expressed on all six days of PC storage. The Fig 2 also shows that the miRNAs hsa-mir-127 (mir127) and hsa-mir-320a (mir320a) are the fourth and fifth most frequently occurring during the first three days of storage, and these miRNAs switch positions on the following three days. Thus, mir320a becomes the 4th most highly expressed miRNA from the 4th to 7th day of storage, whereas mir127 becomes the 8th most highly expressed miRNA. This inverse expression between mir127 and mir320a depending on the storage time may allow us to identify PC bags that still exhibit physiologically normal platelets even after storage more than 5 days (Fig 3A).

### MiRNA expression validation

**Fig 4:**
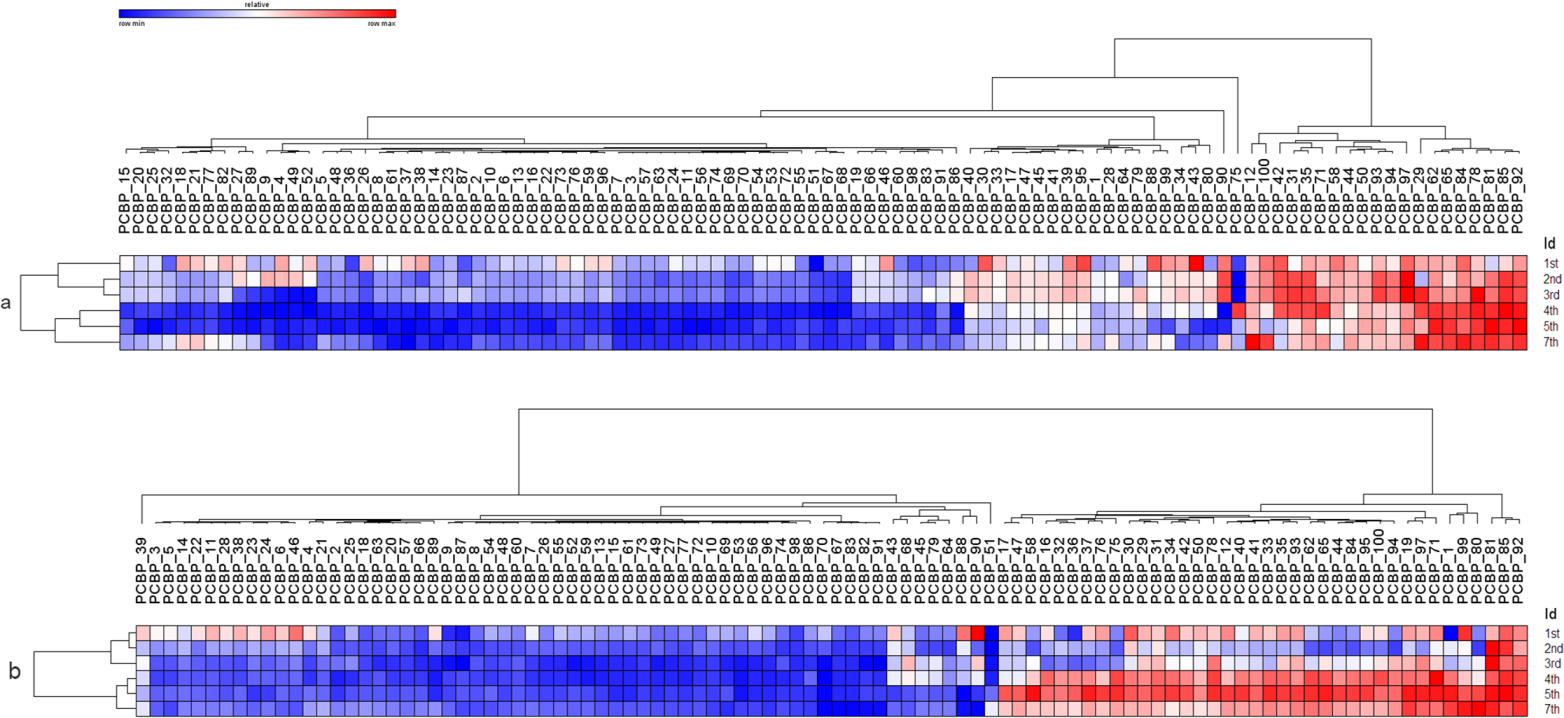
Heatmaps from relative expression of miRNAs, using as a metric the euclidean distance calculated between each PC bag and storage periods for hierarchical cluster construction. Heatmaps was performed with the GENE-E module from GenePattern tool version 3.9.1. The blue bar indicates decreased expression, while the red bar indicates increased expression. (A) A sample of the relative expression of 100 PC bags were hierarchically clustered and scored as statistically significant p <0.001 for six periods of storage, showing that miR-127, was shown to be down-regulated in most PC bags experimentally validated. (B) A sample of the relative expression of 100 PC bags were also grouped hierarchically and scored as statistically significant at p <0.001 during storage periods. This grouping confirmed that miR-320a, was down-regulated in most validated PC bags. In all these 100 PC bags used for the validation of miRNAs (miR-127 and miR-320a), there was a similarity in groups of the 4th, 5th and 7th periods of storage, showing down-regulated profiles that can be related with the biological function of these miRNAs to storage cell damage.

The analyses of sequencing were made on pooled samples from 16 PC. Theoretically this means that increased levels of an individual miRNA could be caused by very high levels in just one or two of the concentrates and may not at all be present in some of the others. To check if this technical artifact happened, and to validate the miRNAs mir127 and mir320a as biomarkers to assess the "validity period" of PC bags stored in blood banks for more than 5 days, 100 PC bags tubes were tested to verify a quantitative relationship between mir320a mir127 in the six different days of storage. The miRNA mir-191 was selected as an internal control because was the miRNA of most highly expressed on six different days of storage. The relative quantification of miRNAs and mir127 mir320a, for mir-191, validated the results found in the Illumina sequencing platform. The inverse expression of these two miRNAs from the 4th day it was confirmed and can be seen in Fig 3A and 3B. In Table 4 we can observe that with increasing storage time also increases the standard deviation of the expression of miRNAs mir127 mir320a and in the heatmaps of the Fig 4A and 4B we can observe the miRNAs expression variation during the storage days in 100 PC bags tested.

**Table 4:** Average expression of miRNAs miR-127 and miR-320a in the six days of storage investigated, their standard errors and *p-value*.

Storage day	Mir-127		Mir-320a	
	*RQ (Mean±SD)	^+^ *p-value*	RQ (Mean±SD)	*p-value*
**1** ^**st**^	0.29 ± 0.06	0.784	0.21 ± 0.05	0.780
**2** ^**nd**^	0.41 ± 0.11	0.850	0.27 ± 0.09	0.815
**3** ^**rd**^	0.42 ± 0.12	0.852	0.38 ± 0.13	0.847
**4** ^**th**^	0.31 ± 0.15	0.787	0.51 ± 0.14	0.876
**5** ^**th**^	0.24 ± 0.19	0.772	0.54 ± 0.17	0.875
**7** ^**th**^	0.36 ± 0.21	0.793	0.60 ± 0.23	0.894

*Real-Time Quantitative PCR

^+^ *p-value* calculated by pair-sample T test.

## Discussion

Platelets help control bleeding by acting as hemostatic plugs in the vascular endothelium, thereby playing an important role in the primary hemostasis of patients [26]. miRNAs are a class of small RNA molecules of approximately 22 nucleotides in their mature state that are involved in regulating gene expression within cells. This regulation occurs through the binding of a miRNA molecule to a messenger RNA by imperfect complementarity in the 3' untranslated region (UTR), thereby interrupting translation and protein production [14].

A study by Landry et al., demonstrated the existence of diverse and abundant miRNAs as key regulators of mRNA translation in human platelets, thus establishing the existence and functionality of a gene regulation process based on miRNA in these anucleate cardiovascular cells [27]. However, there are still no published reports on the characterization of the PC miRnome before or after storage in blood banks.

Because platelets are small, anucleate cells derived from the cytoplasmic fragmentation of megakaryocytes [3], they are unable to synthesize new miRNAs, and therefore, the number of miRNAs is expected to decrease during storage in a blood bank. The half-life of a miRNA molecule varies [28], and miRNAs may suffer rapid turnover [29] or remain stable for longer than 12 days [30]. However, the half-life of platelet miRNAs is expected to be fairly short because platelets circulate in the blood for approximately five days and are then removed from circulation and destroyed by the spleen [31].

PC should be stored between 20°C and 24°C for up to five days under sufficient agitation for proper oxygenation to prevent platelet aggregation [5]. Storage periods depend on local legislation and the additive used in PC solutions, and the validity period is limited by regulations from health authorities to ensure the safety and quality of the product from bacterial growth and the loss of platelet viability during storage due to causes such as lactate accumulation, morphological transformation and platelet aggregation [32].

### Expression Profile of miRNAs in Platelets Stored in the Blood Bank

Approximately 22% of miRNAs were lost from the 1st to 5th day of storage; however, the number of miRNAs increased on the 7th day of storage, which corresponds to the low-quality control for platelets, relative to the 4th and 5th days of storage. This increase was not expected considering the gradual degradation of miRNAs over days in storage. However, it is likely that miRNA precursors (pre-miRNAs) of mature miRNAs that regulate senescence were cleaved after the 5th day of storage by RNA editing enzymes, such as RNase and RNA helicases, thereby increasing the number of identified miRNAs [33]. Another hypothesis that could explain the increase in the number of miRNAs from the 5th to the 7th day assumes that RNA precursors can be cleaved to produce fragments of small RNAs with a regulatory capacity to inhibit protein translation in response to stress [34]. In this case, specifically, the stress would result from aging due to storage for more than five days.

The six lists of the most highly expressed miRNAs contain 14 miRNAs in total; of these, the miRNAs hsa-mir-4433b, hsa-mir-221 and hsa-mir-423 have not previously been associated with platelet physiology. The miRNA hsa-mir-4433b is a precursor of the miRNAs hsa-mir-4433b-3p and hsa-mir-4433b-5p [35]; the expression of miRNAs hsa-mir-221 and hsa-mir-423 were mainly associated with cancers of the lungs [36] and liver [37], respectively, prior to the present study. Expression levels for the other 11 miRNAs (hsa-let-7b, hsa-let-7d, hsa-let-7i, hsa-let-7 g, hsa-mir-22, hsa-mir-28, hsa-mir-92a-1, hsa-mir-99b, hsa-mir-127, hsa-mir-191 and hsa-mir-320a) were reported in published studies on platelets [38–43]; however, some of these miRNAs are also involved in other cellular processes such as aging [44], control of the cell cycle [45] and bodily reactions such as the immune response [46] and inflammation [47,48].

The miRNA hsa-mir-191 was the most highly expressed on all six days of PC storage. At the other extreme, three miRNAs appeared only once on the list of the 10 most highly expressed miRNAs, all in tenth place: hsa-mir-99b, hsa-mir-4433b and hsa-mir-28. The list of the highly expressed miRNAs was somewhat different from previous reports, such as mir-223 [42, 47, 49] probably the analyses of sequencing that were made on pooled samples from 16 CP and the storage time of platelets in the blood bank, where first RNA extraction was performed after 24 hours of storage, may have induced a slight variation.

### Biomarkers to assess the validity of platelet concentrate

The applicability of this study is to indicate biomarkers that can be used to assess the validity of platelet concentrate. An example of this can be observed in inverse expression between mir127 and mir320a that depending on the storage time may allow us to identify PC bags that still exhibit physiologically normal platelets (not activated) even after long storage periods (more than 5 days) that could be used for blood transfusions in the event that fresher bags are unavailable. To our knowledge, there is no report in the literature indicating a direct relationship between the miRNAs mir-127 and mir-320a to mechanisms of platelet activation and/or apoptosis. As previously mentioned, after five days of storage in a blood bank, all unused PC bags are discarded [23]; however, many of these bags may contain functional platelets because the kinetics of cellular aging are influenced by age, type of nutrition and exposure to environmental agents of the blood donors [50,51].

Our example suggests the use of the miRNAs mir127 and mir320a as biomarkers to assess the "validity period" of PC bags stored in blood banks for more than 5 days. Thus, bags can be tested on the 5th day of storage for the relative expression levels of mir127 and mir320a. Expression levels of mir127 lower than those of mir320a would indicate that platelets suffered aging *in vitro* and most likely exhibit storage damage, which would make the PC bag unsuitable for transfusion because this is the PC profile after the 4th day of storage. However, expression levels of mir127 greater than or equal to those of mir320a would indicate that the PC bag still contains physiologically normal platelets because this is the PC profile during the first three days of storage. Validation of the expression of miRNAs mir127 and mir320a, in 100 PC bags tubes, confirmed the findings of Solexa Sequencing. Comparing Fig 3A with Fig 3B we can observe a similarity in the expression of these two miRNAs profile. The pattern of the relative amount of expression of miRNAs mir320a and mir127, and deviation, increase with increasing storage time of platelets in blood bank (Table 4), was due to each of the 100 PC bag is comprised of platelets from different donors and reinforces our hypothesis that miRNAs could be used as biomarkers of "Validity Period" of PC bags stored in blood banks. For example the fifth day of storage can be observed in the standard deviation of Fig 3B that some bags PC tube had a expression levels of mir127 greater than or equal to those of mir320a would indicate that the PC bag still contains physiologically normal platelets. Parameters as swirling, volume of the platelet concentrate, WBC count, platelet count and pH, endorsed this assumption. In future, the assessment of the platelet mitochondrial membrane potential using rhodamine 123 as a probe for mitochondrial membrane potential can confirm our hypothesis. Anyway, the bioinformatics analysis of the target gene prediction of the mir127 and mir320a using the miRWalk Database 2.0, and DAVID Bioinformatics Resources 6.7 for gene enrichment, showed us that these microRNAs regulates several genes of mitochondrial energy metabolism, opening a wide field of research in this area.

Interindividual differences among blood donors is reflected in the different degradation times of miRNAs and show that the use of miRNAs as biomarkers of aging platelets in the blood bank is a promising method. Detecting platelet activation can allow the early detection of changes that affect platelet viability during storage. To conclude that the use of miRNA will contribute to improved quality of blood products the results need to be correlated to morphological marker of platelet storage lesion. The unique morphology of platelets and certain functional characteristics can be assessed by flow cytometry, which uses monoclonal antibodies to recognize changes in the expression levels of glycoproteins on the surface of platelets that act as pro-coagulants [52,53]. However, the main purpose of detecting platelet activation during PC preparation and storage is to assess the final quality of the PC bag and allow the selection of PC bags that have been stored for more than 5 days but are still suitable for blood transfusion.

## Conclusion

The firm conclusion that can be drawn from the data is that platelet concentrates contains miRNA and the concentrations varies over time. To our knowledge, there is no method to identify PC bags with functional platelets among those to be discarded after five days of storage in blood banks. Thus, evaluating the ratio of the expression levels of mir127 and mir320a (mir127/320a) may become a unique and extremely important methodology for transfusion medicine because it can be performed quickly before PC bags are discarded. However, further studies are needed.
